# The deposition of thin films of cadmium zinc sulfide Cd_1−*x*_Zn_*x*_S at 250 °C from spin-coated xanthato complexes: a potential route to window layers for photovoltaic cells

**DOI:** 10.1007/s10853-017-1872-1

**Published:** 2017-12-07

**Authors:** Ali A. K. Bakly, Ben F. Spencer, Paul O’Brien

**Affiliations:** 1 School of Materials, University of Manchester, Oxford Road, Manchester, M13 9PL UK; 2 School of Chemistry, University of Manchester, Oxford Road, Manchester, M13 9PL UK

## Abstract

Thin films of Cd_1−*x*_Zn_*x*_S (CZS) were prepared by a novel spin coating/melt method from cadmium ethylxanthato [Cd(C_2_H_5_OCS_2_)_2_] and zinc ethylxanthato [Zn(C_2_H_5_OCS_2_)_2_] in *x* ratios of 0–0.15 and of 1. A solution of the precursor(s) in THF was spin coated onto a glass substrate and then heated at 250 °C for 1 h under N_2_. The thickness of the film formed can be controlled by varying the solution composition and/or the spin rate of the coating. A total metal precursor solution concentration of 50 mM was used in all cases. The films were characterized by p-XRD, SEM, EDX, ICP-AES, XPS, UV–Vis absorption spectroscopy, Raman spectroscopy and resistivity measurements. The band gaps of the films were between 2.35–2.58 and 3.75 eV (0 ≤ *x* ≤ 0.15 and at *x* = 1). The resistivity of Cd_1−*x*_Zn_*x*_S films was found to vary linearly with zinc contents, and the properties of the films suggest potential application to photovoltaics as window layers. This work is the first study to demonstrate Cd_1−*x*_Zn_*x*_S thin films by a spin coating/melt method from xanthato precursors.

**Electronic supplementary material:**

The online version of this article (10.1007/s10853-017-1872-1) contains supplementary material, which is available to authorized users.

## Introduction

Thin films of Cd_1−*x*_Zn_*x*_S are interesting because their properties lie between those of ZnS and CdS [1–5]. It is considered a relatively useful transparent metal sulfide, for use as a window in photovoltaic cells. It has a lower absorbance in the UV with band gaps typically in excess of ~ 2.4 eV [1, 5–8]. There is also a relatively small lattice mismatches to CdS/CdTe or CIGS cells (Supporting Information; Table S6 and Figure S13) [9–12]. Such materials also establish electric fields at the heterojunction interface and may act as a buffer layer [5, 12]. The resistivity should be high enough to reduce the trap state density at the interface of the buffer and absorber layers, to improve the junction properties [10].

Thin films and nanostructures properties are sensitive to the preparation method used and synthetic route [13–15]. Numerous techniques have been used to grow CdS and Cd_1−*x*_Zn_*x*_S thin films including dip coating [16], electrodeposition [17], chemical vapor deposition (CVD) [18], aerosol-assisted CVD (AA-CVD) [19–21], chemical bath deposition (CBD) [2, 8, 22, 23], spray pyrolysis [24, 25], thermal evaporation [26], successive ionic layer and reaction (SILAR) [27], thermolysis [28, 29], electrochemical atomic layer epitaxy (ECALE) [11], sol–gel spin coating, [30, 31] doctor’s blade, [32] and others [14, 21, 33, 34]. Spin coating (SC) is a particularly useful method for the deposition of thin films [35, 36]. A melt of precursors provides a novel low-temperature route to metal chalcogenides; it has good atom efficiency in a high yield of 100% Zn metal as compared to AA-CVD or CBD [37]. In the present work, a metal chalcogenide precursor solution in tetrahydrofuran (THF) is coated onto a glass substrate. The process is repeated until the desired thickness of the chalcogen film has been formed. The final thickness of the film is determined by the precursor used, the concentration of the precursors, the solvent viscosity and the spinning parameters (speed, time). In a related CBD study, Ampong et al. [1] reported a ternary alloy with a hexagonal (wurtzite) structure and a wider band gap than a CdS film [2, 8]. The two most common phases for CdS and ZnS are the well-known hexagonal (greenockite) CdS and the cubic zincblende (sphalerite) [38].

Wurtzite stacking sequence expressed as ABABABAB pattern along the “close-packed” c-axis, whereas the stacking sequence of zincblende expressed as ABCABCABC pattern along the same [111] direction perpendicular to the sulfur planes. Moore et al. [39] determined that the two phases of ZnS are quite similar in structure and can be transformed simply by changing the stacking sequence. Cadmium sulfide is more stable as the hexagonal (wurtzite structure). Singh et al. noted that wurtzite ZnS and CdS structures have cation radii in the ratio of Zn^2+^/Cd^2+^ ≈ 0.77 [40].

In this study, we report the thermal decomposition of cadmium ethyl xanthato [Cd(C_2_H_5_OCS_2_)_2_] (1) and zinc ethylxanthato [Zn(C_2_H_5_OCS_2_)_2_] (2) as a single molecular precursor (SMPs) for deposition of CdS, ZnS and Cd_1−*x*_Zn_*x*_S thin films by spin coating. The films, ternary alloys Cd_1−*x*_Zn_*x*_S, were prepared in the range of mole percentage of Zn from 0 to 15 and at 100%. The optical, electrical and crystalline properties of the deposited films correlate linearly with the mole percentage of zinc content. The CdS and CdZnS films are hexagonal, while the pure ZnS film is a cubic film. The band gap for CdS thin film is 2.35 eV and varies linearly up to 2.55 eV at 15 mol% Zn; the ZnS band gap is 3.75 eV, agreeing well with the literature band gap values of 2.39 and 3.75 eV for CdS and ZnS, respectively, at room temperature. Ampong et al. [1] concluded that the optical band gap mirrors the structural changes in Cd-rich samples, notwithstanding overall the introduction of small quantities of Zn, which leads to a systematic shrinkage of the hexagonal lattice (up to 12% Zn).

## Experimental

### Materials and instrumentation

All preparations were carried out on a Schlenk line under a dry nitrogen gas stream. All chemicals were used without any purification and were from Sigma-Aldrich or Fisher. The elemental analyses (EA) were carried out with a Flash 2000 thermo-scientific elemental analyzer and using a Thermo iCap 6300 Inductively Coupled Plasma Atomic Emission Spectroscopy (ICP-AES). Thermogravimetric analysis (TGA) measurements were taken using a Seiko SSC/S200 model at a heating rate of 10 °C min^−1^ from 30 to 600 °C under nitrogen. The optical absorption spectra (UV–Vis) were recorded at room temperature with a Shimadzu double-beam UV-1800 spectrophotometer in the wavelength range of 800–300 nm at 1 nm resolution. Grazing incidence powder X-ray diffraction (p-XRD) patterns were obtained at room temperature using a Bruker D8-Advanced diffractometer with the range of 15°–65°, step size of 0.050° and a dwell time of 8 s, using [Cu Kα radiation source (λ = 1.5418 Å), 40 kV, 40 mA] with X’Pert High Score Plus software. Scanning electron microscopy (SEM) measurements were taken using a Philips XL30. The elemental composition of the samples was determined using an energy-dispersive X-ray spectroscopy (EDX) connected to the scanning electron microscope unit. Before carrying out the SEM and EDX, carbon coated of 11.5 nm thickness was applied by a Quorum Model Q150T-ES precision coating system. The sheet resistivity was determined using a Keithley 2614 type general purpose source meter employing the four-point probe method. X-ray photoelectron spectroscopy (XPS) was carried out to obtain chemical state information using a Kratos Axis Ultra DLD and the data fitted with Gaussian–Lorentzian convolutions using the Casa XPS software (www.casaxps.com).

### The synthesis of cadmium ethyl xanthato [Cd(S_2_COCH_2_CH_3_)_2_] (1)

The cadmium ethylxanthato [Cd(S_2_COEt)_2_] was prepared by a method reported in the literature [3, 41]. A solution of potassium ethylxanthato KS_2_COC_2_H_5_ (4.9 g, 31.2 mmol) in 50 ml of distilled water was added dropwise to an aqueous solution of cadmium chloride (2.8 g, 15.6 mmol) dissolved in 25 ml of distilled water, with continuous stirring for 15 min. A creamy-white, yellowish, precipitate formed immediately, which was filtered, then washed with distilled water twice and then dried under vacuum at 25 °C for 6 h. The cadmium ethyl xanthato compound [Cd(S_2_COEt)_2_] was purified by recrystallization using ethyl acetate as a solvent with gentle heating. Yield 6.9 g (87.82%) and melting point (mp) 166 °C. EA (%), calculated (found): C, 20.32 (20.31); H, 2.84 (2.79); S, 36.09 (36.31); Cd, 31.72 (31.24). FTIR absorption signals (cm^−1^): 2985 (m), 2162.45 (n), 1469 (m), 1390.46 (m), 1365.43 (w), 1270.64 (m), 1179.28 (s), 1117.73 (s), 866.51 (m), 817 (m). ^1^H NMR chemical shift: (400 MHz, DMSO-d_6_) *δ* = 1.30 (*t*, *J* = 7.08 Hz, 3H), 4.33 (*q*, *J* = 7.08 Hz, 2H), (Supporting Information; Figure S14); ^13^C NMR (DMSO-d_6_): 14.49 (CH_3_), 72.76 (CH_2_) ppm).

### The synthesis of zinc ethyl xanthato [Zn(S_2_COCH_2_CH_3_)_2_] (2)

The zinc ethylxanthato [Zn(S_2_COEt)_2_] was prepared according to a modified established method [3, 41]. A solution of potassium ethylxanthato (4.9 g, 31.2 mmol) in 50 ml of distilled water was added dropwise to an aqueous solution of zinc chloride (2.1 g, 15.6 mmol) dissolved in 25 ml of distilled water. A white precipitate formed which was filtered and then washed with distilled water twice and then dried under vacuum at 25 °C for 6 h. The compound was purified by recrystallization using acetonitrile solvent. Yield 5.8 g (81.42%) and melting point 140–143 °C. EA (%), calculated (found): C, 23.42 (23.27); H, 3.28 (3.23); S, 41.61 (41.72); Zn, 21.28 (21.02). FTIR absorption signals (cm^−1^): 2982 (w), 2251.31 (n), 1462.82 (m), 1386.83 (s), 1364.31 (m), 1287 (s), 1202.66 (s), 1155.21 (w), 1119.11 (s), 1029.68 (s), 999.19 (w), 861.39 (w). ^1^H NMR chemical shift: (400 MHz, DMSO-d6) *δ* = 1.26 (*t*, *J* = 7.03 Hz, 3H), 4.33 (*q*, *J* = 7.28 Hz, 2H), (Supporting Information; Figure S15); ^13^C NMR (DMSO-d6): 14.48 (CH_3_), 72.77 (CH_2_) ppm.

### Spin coating method

Samples were prepared by a spin coating method. The precursors (1) and (2) were dissolved in THF with sonication in (0 ≤ *x* ≤ 0.15 and *x* = 1) mol ratios; 3.5 cm^3^ of a solution was used for each deposition with 50 mM at total metal concentration. The clear solution was dropped onto glass substrates by pipette at room temperature. The solution was spin coated on glass substrates at an angular rotation rate of 1500 rpm for 1 min. The samples were then placed in a tube furnace under a nitrogen stream at a flow rate of 20 sccm (cm^3^/min) at 250 °C for 1 h. All samples were cooled at room temperature for 1.5 h under a nitrogen stream.

## Results and discussion

The metal ethylxanthato [Cd(C_2_H_5_OCS_2_)_2_] (1) and [Zn(C_2_H_5_OCS_2_)_2_] (2) are both readily soluble in THF. The total precursor solution concentration was 50 mM and was used to form Cd_1−*x*_Zn_*x*_S films by spin coating, followed by decomposition at 250 °C. The method offers a route to Cd_1−*x*_Zn_*x*_S films of controlled composition and thickness. This technique is suitable for semiconductor fabrication and uses easily prepared precursors.

**Figure 1:**
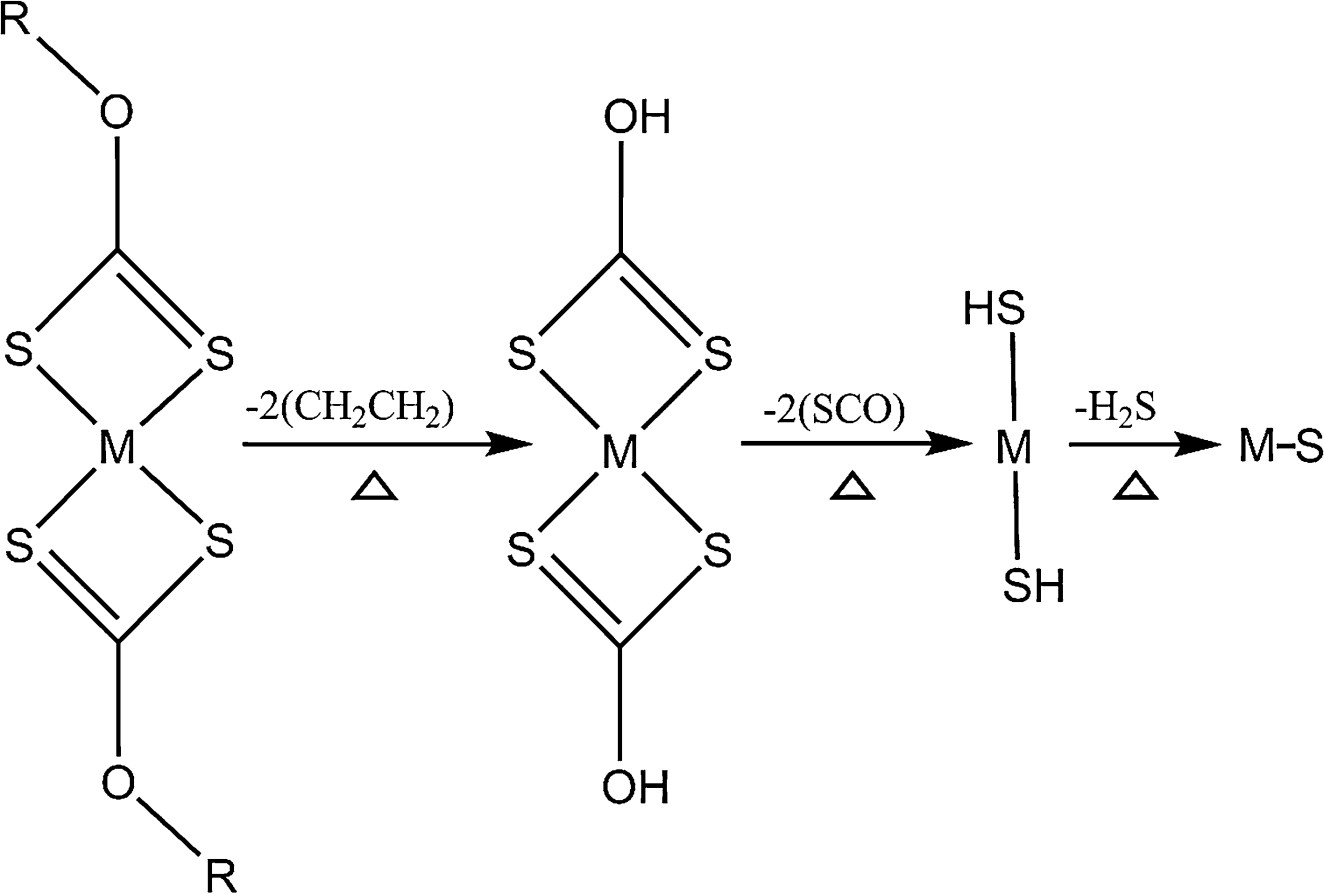
Suggested breakdown of metal ethyl xanthato precursor at 250 °C by a modified Chugaev elimination mechanism [42]

The xanthato precursors are expected to decompose by a modified Chugaev elimination mechanism [42]. The Chugaev elimination mechanism (Fig. 1) suggests that the first step of the decomposition of xanthato compounds results in [M(S_2_COH)_2_] which leads eventually to MS.

**Figure 2:**
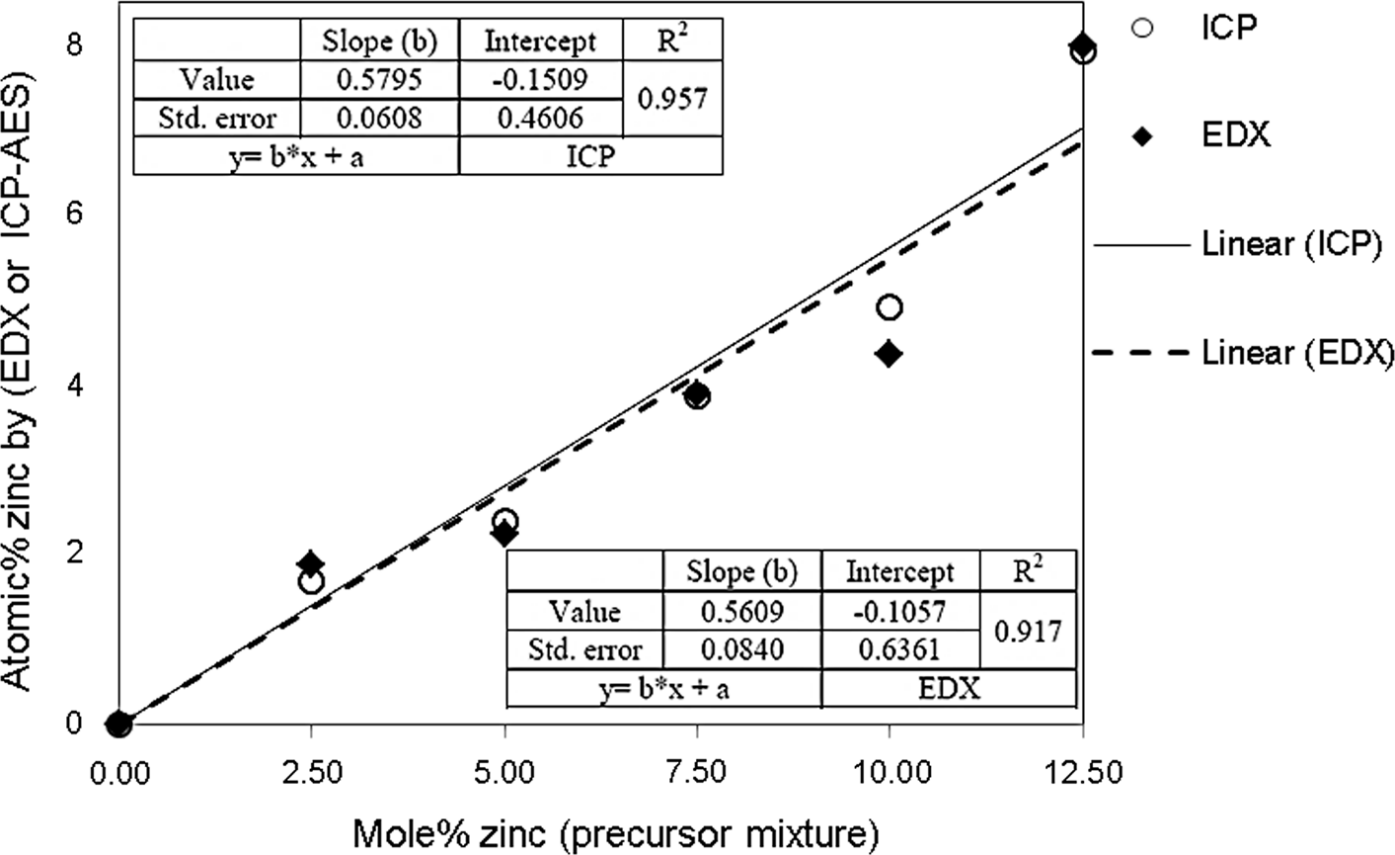
Plot of mol% zinc in the precursor mixture against alloy composition as determined by EDX or ICP-AES (Cd_1−*x*_Zn_*x*_S, 0 ≤ *x* ≤ 0.125) for thin films produced by spin coating the precursors followed by decomposition at 250 °C

Elemental analysis by EDX and ICP-AES of the dissolved films of Zn, Cd and S both confirmed a consistent stoichiometry. The results are summarized in Fig. 2 and tabulated in the Electronic Supporting Information ESI sheet (Supporting Information, Table S1). The plots in Fig. 2 show linearity over the range 0 ≤ *x* ≤ 0.125.

**Figure 3:**
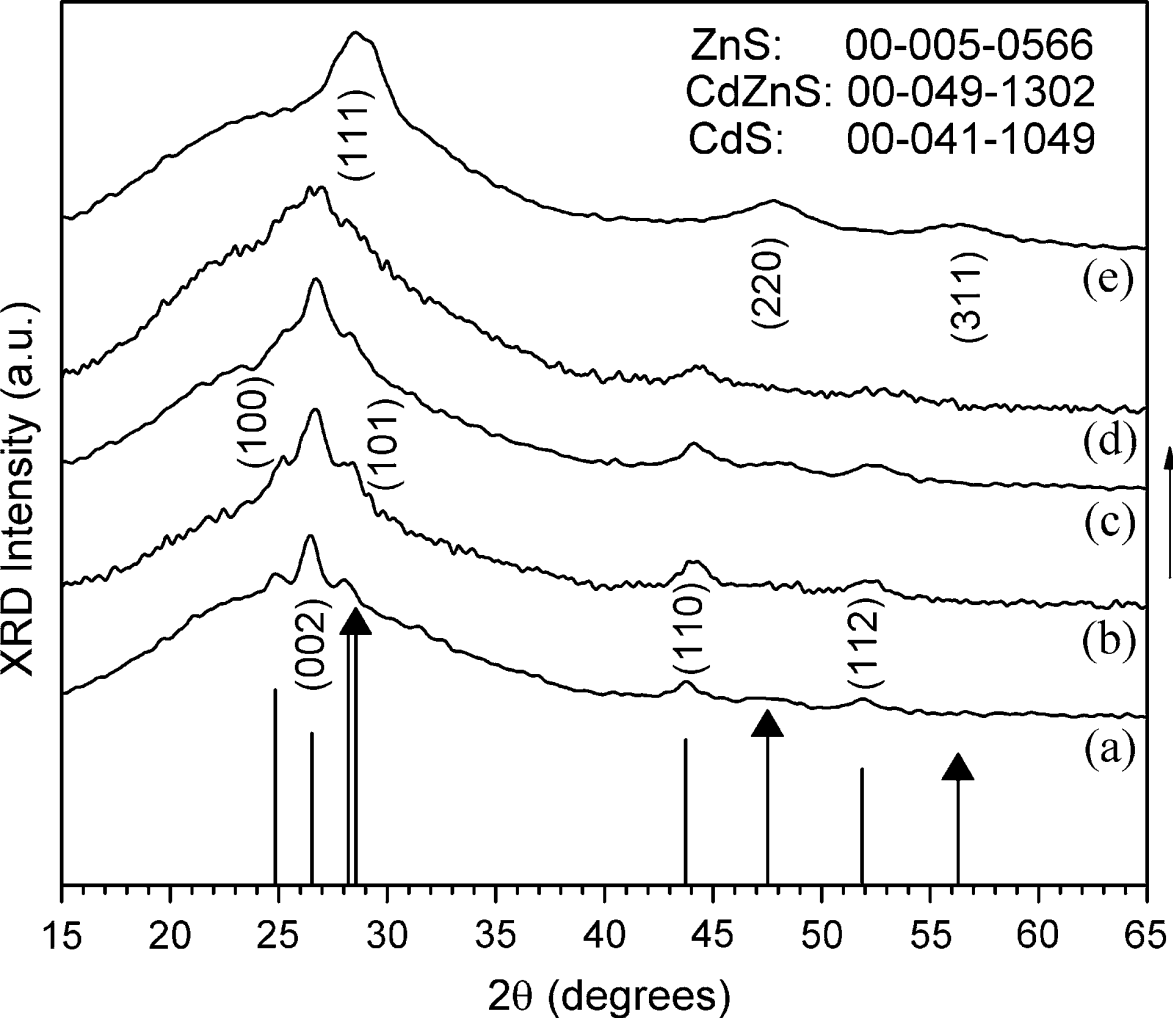
(p-XRD) patterns of Cd_1−*x*_Zn_*x*_S films deposited in the composition range (0 ≤ *x* ≤ 0.15 and *x* = 1). The p-XRD patterns of *a* pure CdS; *b* 7.5 mol% zinc; *c* 12.5 mol% zinc; *d* 15 mol% zinc; and *e* pure ZnS thin films are shown. The intensities and positions of the standard peaks for CdS and ZnS are indicated. The lines capped with triangles correspond to reflections from the cubic phase of ZnS while the others as hexagonal CdS

Studies by p-XRD showed that thin films formed at doping ratios between (0 ≤ *x* ≤ 0.15) were hexagonal. The p-XRD patterns of the deposited films at different compositions are shown in Fig. 3. The peaks of CdS corresponding to the reference pattern (JCPDS, reference code: 00-041-1049) are shifted to higher angles as the zinc content increases. The sequential shifts of the p-XRD patterns confirm that the crystals are Cd_1−*x*_Zn_*x*_S and not a mixture of ZnS and CdS. The major peaks can be assigned to the (100), (002), (101), (110) and (112) reflections. All patterns were indexed, and unit cell parameters are detailed in the supplementary sheet (Supporting Information; (Equation a), Table S2, and Figures S3 and S8). The deposited films give reasonable diffraction patterning p-XRD. The structure of the ZnS film deposited by this method is cubic, ZnS cubic pattern and standard peaks shown in Fig. 3. Earlier reports have reviewed a nanostructured film with Cd_0.9_Zn_0.1_S hexagonal and Cd_0.5_Zn_0.5_S polytypic or cubic form existing in the hexagonal phase, while Cd_0.1_Zn_0.9_S shows the cubic phase [1, 2, 43].

X-ray photoelectron spectroscopy (XPS) was used to measure the CdS and ZnS films that were annealed at 250 °C. The binding energy (BE) positions of the Cd 3*d*, Zn 2*p* and S 2*p* core level photoelectrons are attributed to CdS and ZnS as expected [44, 45]. The XP spectra showed a single chemical species for Cd with Cd 3*d*_5/2_ at 405.2 eV with a spin–orbit splitting of 6.8 eV as expected for the  + 2 Cd state (Supporting Information; Figure S10) [44]. The Zn 2*p* spectrum shows a single chemical species with a 2*p*_3/2_ peak at 1022.2 eV binding energy with a spin–orbit splitting of 23.2 eV, associated with ZnS (Supporting Information; Figure S11) [45]. The S 2*p*_3/2_ core spectrum was measured with a peak at a binding energy position of 161.6 eV, associated with CdS and ZnS with the same electronegativity (Supporting Information; Figure S12) [44, 45].

None of the diffraction patterns shows evidence for the formation of crystalline CdO, which was also evidenced by the films being well defined, smooth and transparent yellowish in all cases.

Cubic zincblende “sphalerite” is the more stable phase at lower temperatures [14, 19, 33, 46]. Ramasamy et al. reported that the structure of ZnS is temperature dependent; the deposited films were cubic ZnS at 300 and 350 °C, while at 400 and 450 °C hexagonal ZnS with granular crystallites predominant [19]. Zhao et al. found that synthesized ZnS by colloid chemistry usually gives a stable phase of a cubic sphalerite structure at low temperatures. The wurtzitic phase typically forms at a temperature greater than 1023 °C (1296 K) [47]. Ramasamy et al. reported that the XRD pattern of ZnS films displays a cubic-to-hexagonal phase conversion above 350 °C [46]. The p-XRD of ZnS structure (Fig. 3) shows broad peaks corresponding to sphalerite. Barnes et al. [48] first suggested sulfur fugacity (*f*S_2_) is a key in determining the phase form. Low sulfur fugacity decreases the transition temperature to ca. 500 °C, while for a higher fugacity of sulfur the transition temperature moves to above 1000 °C. Wold et al. [49] determined that the growth of hexagonal ZnS thin films generally occurs at higher temperatures. The cubic ZnS film can form at high temperatures on closely lattice-matched substrates.

The surface morphology of thin films is important for applicability and has a strong influence on the optical properties of the films. Abdelhady et al. [29] found a major influence on surface morphology that happens by the precursor concentration, growth temperature and reaction time, while the optical properties of the particles are strongly dependent on the ratio of the ZnS to CdS in the feed solution. The SEM images confirm that the effect of low Zn doping content changes the microstructure of Cd_1−*x*_Zn_*x*_S thin films.

**Figure 4:**
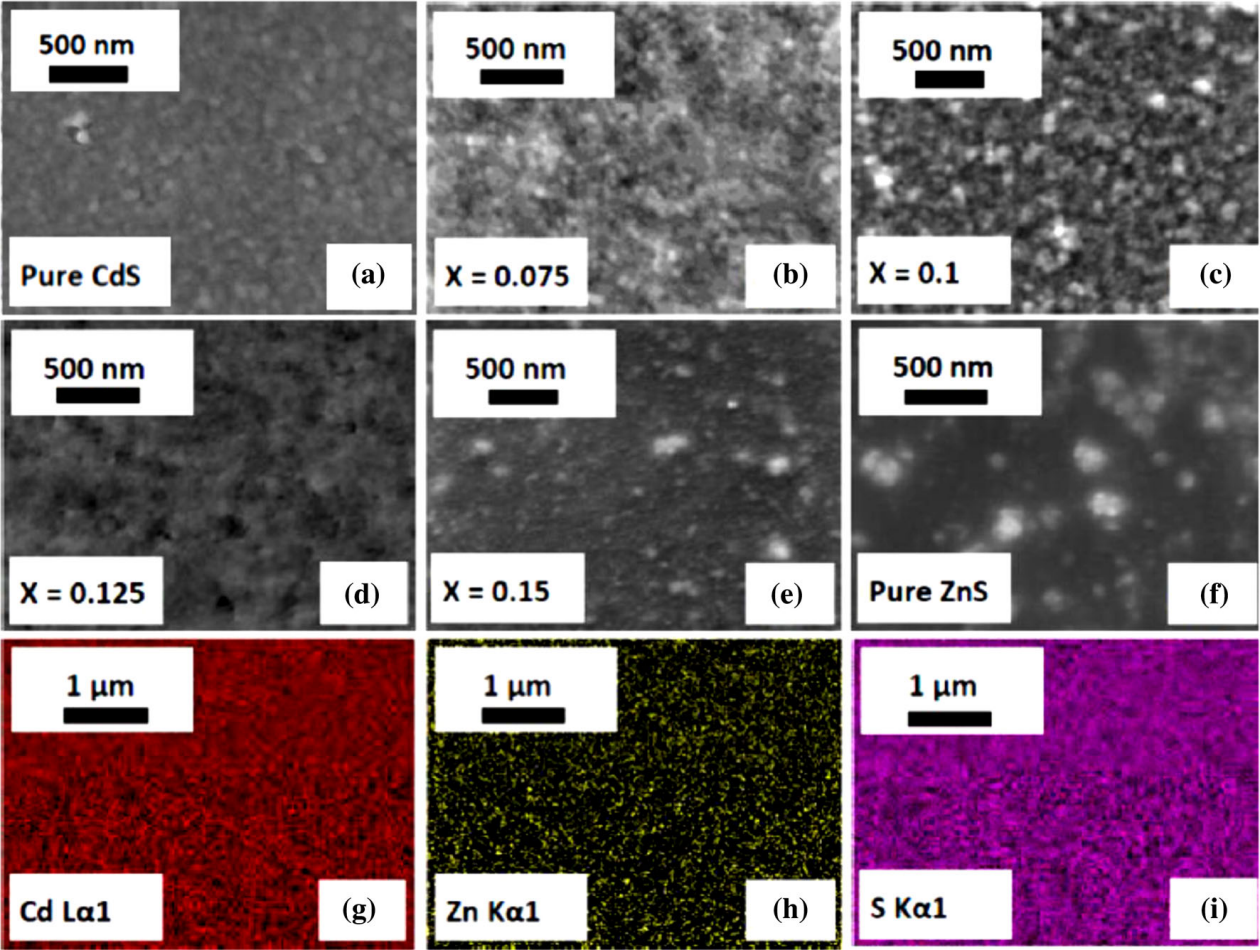
Secondary electron microscopy SEM images (10 kV) of Cd_1−*x*_Zn_*x*_S films deposited in the composite range (0 ≤ *x* ≤ 0.15 and *x* = 1); the images: **a** pure CdS; **b** 7.5 mol% zinc; **c** 10 mol% zinc; **d** 12.5 mol% zinc; **e** 15 mol% zinc; and **f** pure ZnS thin films. (All magnification scales are 500 nm.) EDX spectrum mapping of the elemental distributions (20 kV) of Zn, Cd and S (colored images); **g** Cd Lα1, **h** Zn Kα1 and **i** S Kα1 emission at 12.5 mol% zinc-doped CdS films. (All EDX scale bar corresponds to 1 μm.)

A smooth surface leads to transparency and enhanced light transmission, whereas irregular films scatter light. Significant research has been conducted, including Lee et al. [50] where it was demonstrated that a non-smooth surface could cause light scattering. SEM images of the as-deposited films reveal the presence of compact films and smooth grains, and therefore, an enhanced transmission efficiency is accomplished (Fig. 4). Kamuruzzaman et al. [51] demonstrated that increasing the annealing temperature leads to an improved surface homogeneity, and the crystallinity increases due to Cd diffusion over the surface which reduces defects and surface roughness. Increasing the annealing temperature of the films leads to the amorphous phase diminishing, since more energy is supplied for crystallite growth. Increasing the zinc content of the films improves the surface morphology and microstructure. SEM confirms that even low zinc content changes the microstructure of Cd_1−*x*_Zn_*x*_S films. Binary and ternary alloys often obey Vegard’s law, with a linear relationship between the lattice constants and composition. This is often valid for substituted solid solutions such as CdZnS [1, 52, 53]. Peter et al. found that a gradual decrease in lattice parameters is subject to the Zn content increases and that increasing Cd concentration in the film leads to the grain size increasing [53].

The optical properties of the films were measured over the wavelength range 300–800 nm at room temperature. The optical density at the absorption edge did not exceed 0.55 in all cases. A plot of (*αhν*)^2^ versus hν was plotted for different zinc compositions in order to estimate the optical band gap and transition type of the films from the Stern relation for near edge absorption (Supporting Information; Equation b) [54]. The band gap energy was obtained by extrapolating the linear portion of (*αhν*)^2^/*n* versus *hν* to the energy axis. A straight line at higher energies indicates a direct optical transition for the majority of II–VI compounds, and hence, the *n* value of 1 is assumed (Supporting Information; Equation b).

**Figure 5:**
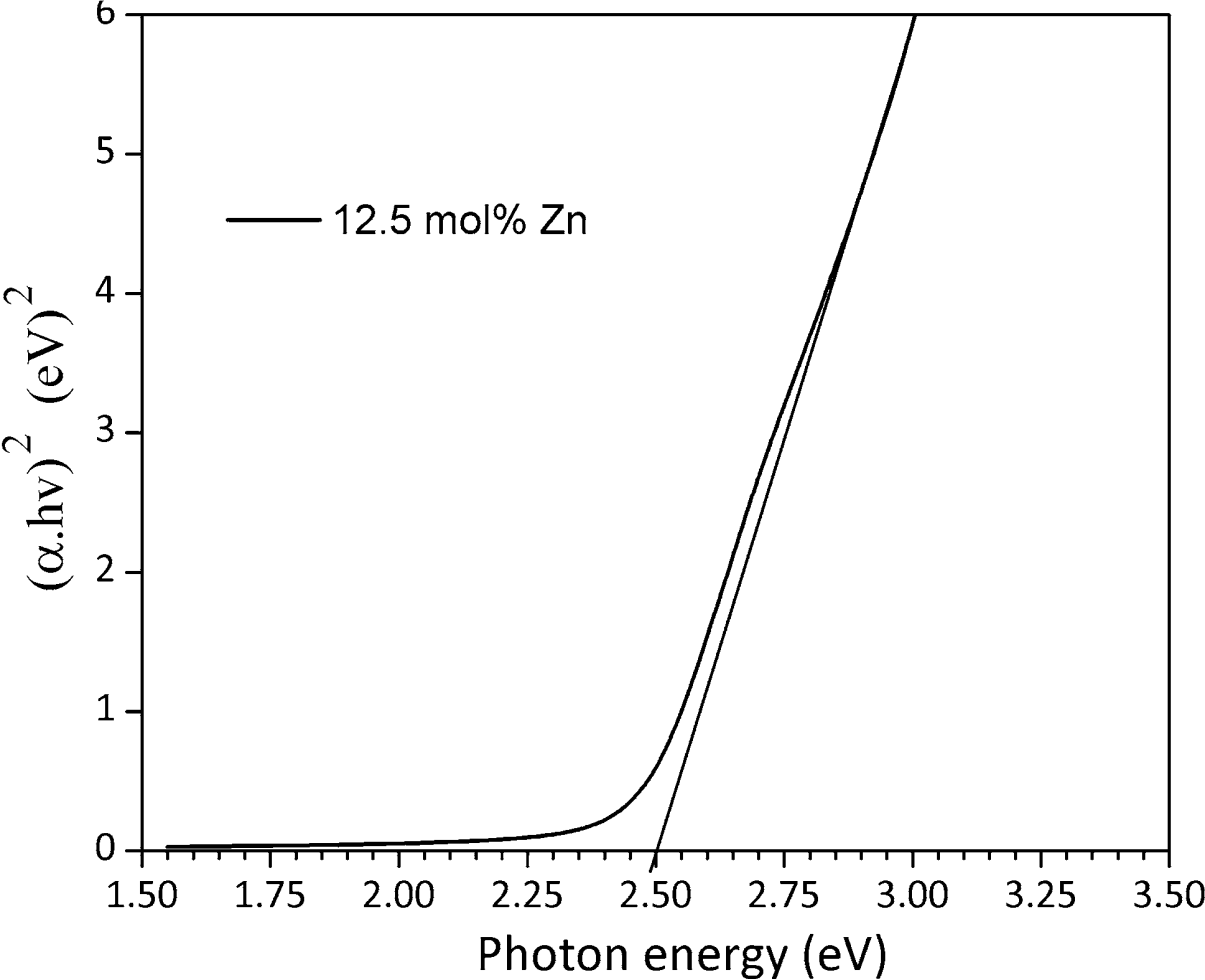
A typical Tauc plot showing extrapolation of the absorption spectrum to obtain the optical band gap for 12.5 mol% zinc film deposited from (1) and (2) spin coated on a glass substrate (250 °C for 1 h) under N^2^ gas stream (Supporting Information; Table S4)

Alloying Cd_1−*x*_Zn_*x*_S allows a systematic variation in the band gap of Cd_1−*x*_Zn_*x*_S with composition [55]. The behavior is in accordance with the Burstein/Moss shift [56], which describes the shift in doped samples to higher energies of the band gap due to an increase in the carrier concentration. The band gap of CdS film is 2.35 eV. The alloy in the range (2.5–15) mol% of Zn content allows for the band gap to be tuned between 2.38 and 2.55 eV with doping (Supporting Information; Figure S16). Figure 5 shows a plot of (*αhν*)^2^ versus hν for Cd_1−*x*_Zn_*x*_S thin film at zinc concentration of 12.5 mol% Zn with a linear variation in the band gap.

Band gap defects are often electrically active and introduce energy levels within the band gap. The origin of the defect states is related to cadmium and zinc vacancies, which are shallow donor levels [7, 57, 58]. Defect states change the electrical properties of the films, observed as a sharp increase in the band gap, and a single optical gap was observed. A band gap for zinc sulfide of 3.75 eV was measured as expected for bulk ZnS (Supporting Information; Figure S17) [1].

**Figure 6:**
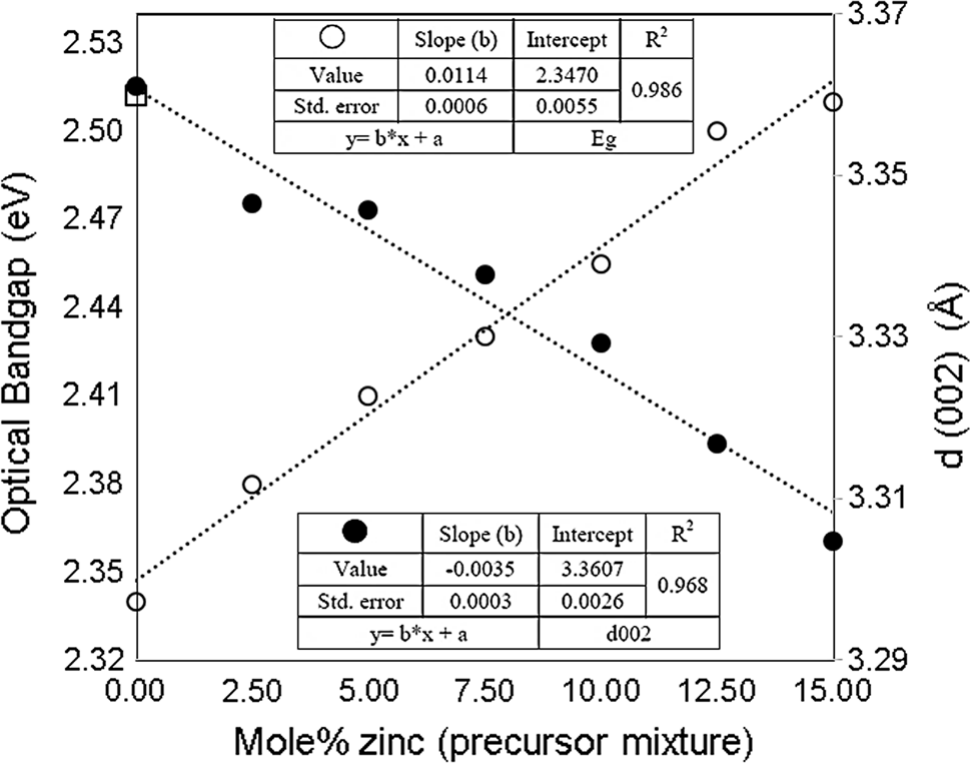
Mol% zinc in precursor mixture against optical band gap and d-exp (002) (Cd_1−*x*_Zn_x_S, 0 ≤ *x* ≤ 0.15) of thin films produced by spin coating precursors (1) and (2) followed by decomposition at 250 °C for 1 h. The literature d-spacing (002) value of the CdS 2H-wurtzite structure 3.360 Å [2]

ICP-AES analysis showed that zinc content increased over the doping and that the molar changes in the zinc percentage are reflected in the changes in structural and optical properties which linearly mirror each other over the range (0 ≤ *x* ≤ 0.125) (Fig. 6).

**Figure 7:**
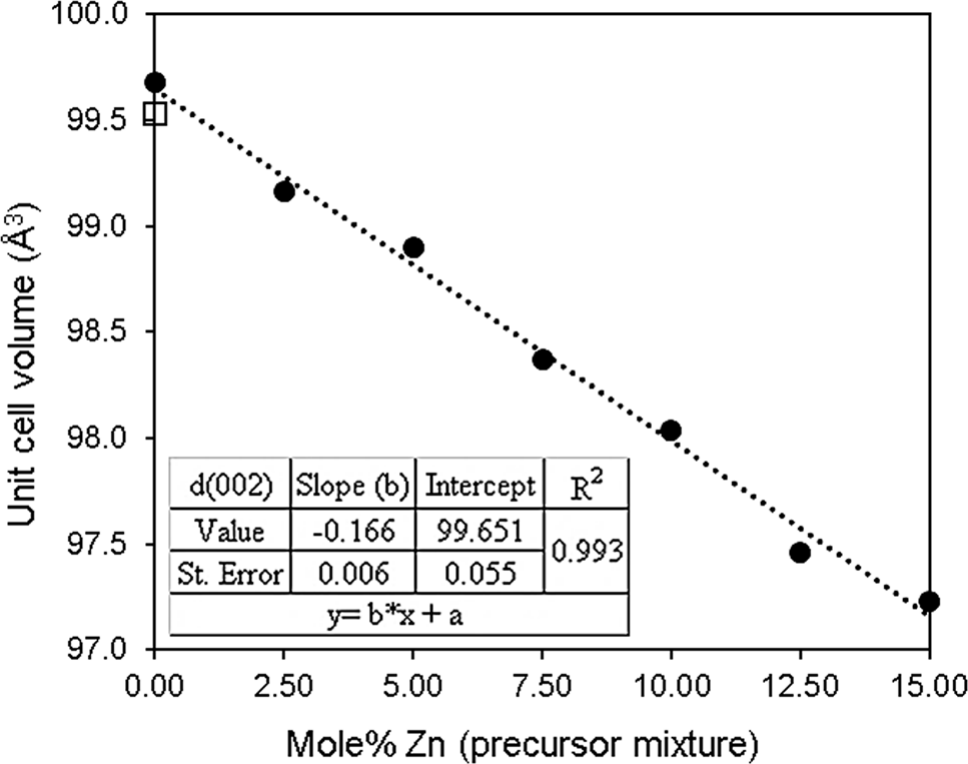
Mol% zinc in precursor mixture against unit cell volume (Cd_1−*x*_Zn_*x*_S, 0 ≤ *x* ≤ 0.15) of thin films produced by spin coating, followed by decomposition at 250 °C for 1 h, lattice parameters were measured by p-XRD. Literature value of crystalline wurtzite-2H CdS is 99.53 Å^3^, Yeh et al. [38]

As the zinc contents increase (2.5 to 15%), the c/2 (002) reflection decreases, as may be expected due to the lattice contraction along the [001] direction (Fig. 6). Wurtzite CdS usually grows along the [001] direction (c-axis) [1]; the c-axis is energetically preferred for the growth/relaxation of the wurtzite lattice [59]. At mol% of Zn greater than 12%, the lattice contraction becomes uncorrelated [1]. The systematic variations in the lattice constants (a and c) (Supporting Information; Table S2 and Figure S3) are consistent with the substitution of tetrahedrally coordinated Zn^2+^ (ionic radius = 0.74 Å) into the hexagonal CdS (rCd^+2^ = 0.97 Å) [60, 61]. A gradual linear decrease in lattice parameters (a and c) with increasing zinc composition was observed in the range up to 12.5 mol%. Figure 7 shows the variations of unit cell volume (Å^3^) that are linear with the Zn content, associated with defects in the lattice [62].

The structure is hexagonal with a smooth variation of the lattice parameters up to 15 mol% of Zn [1]. Linearity ceases at this level of doping and is followed by an abrupt change to cubic ZnS at Zn > 75%. The yellow color of the films lightens as the zinc content increases. The zinc-containing films are smoother and less light scattering.

**Figure 8:**
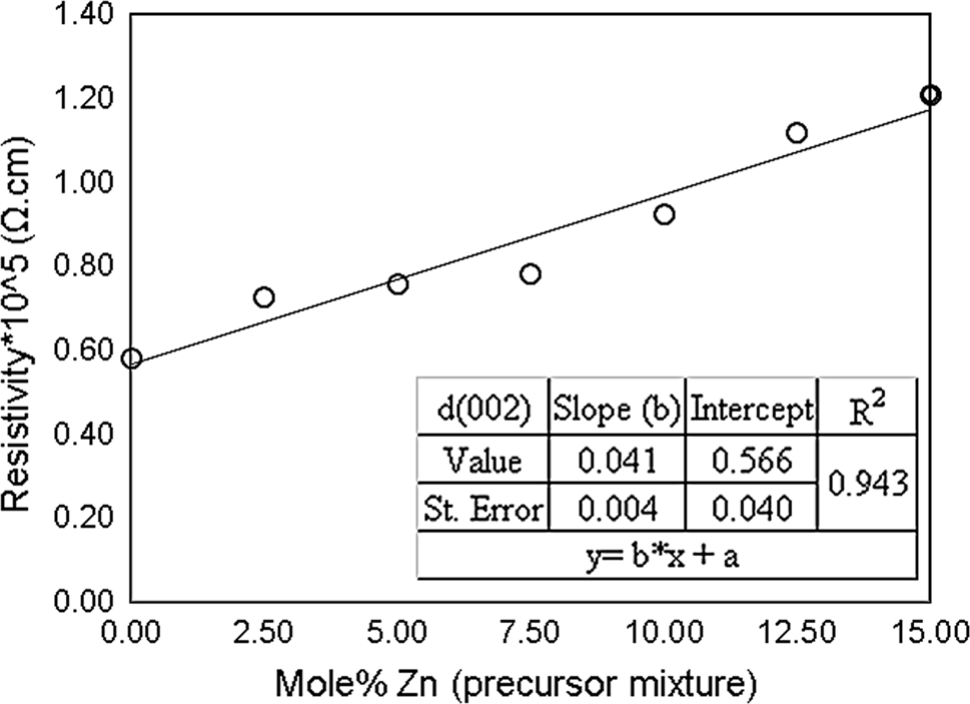
Mol% zinc in precursor mixture against the measured resistivity (Cd_1−*x*_Zn_*x*_S, 0 ≤ *x* ≤ 0.15) of thin films produced by spin coating precursors (1) and (2) followed by decomposition at 250 °C for 1 h. The literature values are in the ranges 10^2^–10^8^ Ω cm [66] and 10^3^–10^5^ Ω cm [67]

A wider band gap window material is useful for applications in heterojunctions of photovoltaic cells, and a low resistivity less than 10^2^ Ω cm is generally required [63], and here the sheet resistance is often linearly dependent on the zinc content [64, 65]. The electrical resistivity of CdS films decreases with film thickness [66] and decreases to 10^5^ Ω cm as the temperature is increased (200–400 °C) [67]. The electrical resistivity and surface defects are strongly dependent on the deposition method [66]. In this study, the resistivity measurement was taken using the four-probe technique; the current was transferred through the outer ends and the likely drop measured across the other two inner ends. The resistivity of the films was found to increase linearly in the range of 0 ≤ *x* ≤ 0.15, (Fig. 8) (Supporting Information; Table S5 and Figure S9). The resistivity can be reduced considerably by increasing the film thickness, which can be attributing to the effect of crystallite size on carrier mobility. Al Kuhaimi [66] referred to the reversible correlation between the resistivity and film thickness and that this was related to the stoichiometry of the film, the effect of the crystallite size, the degree of preferred orientation and internal microstrain. Further work is therefore required to make these films applicable to photovoltaics; now the film deposition method has been well characterized.

Sheet resistance is a one-dimensional entity; as with the band gap, it is linearly dependent on zinc doping. The electrical resistivity of Cd_1−*x*_Zn_*x*_S films in CdTe devices increases in the range 1–10^10^ Ω cm with the zinc content (0–1) [10].

## Conclusion

Thin films of CdS, Cd_1−*x*_Zn_*x*_S and ZnS have been deposited from xanthato complexes by spin coating the precursor(s) onto glass substrates, followed by thermal decomposition. Zinc-doped CdS thin films were deposited at different molar ratios of the precursors. At lower doping levels *x* ≤ 0.125, a linear variation in structural, optical and electrical properties is observed. The thin films had a range of morphologies, measured by SEM. The films become smoother and less scattering and are more transparent as the zinc content is increased, and the band gap increases from 2.35 eV (CdS) to 2.55 eV at 15 mol% of Zn. The lattice of CdZnS mismatch with common semiconductors lies in the range 0.03–9.5 %, (Supporting Information Table S6 and Figure S13). The method is potentially useful for window layers in CdS/CdTe and CIGS photovoltaic cells.

## Electronic supplementary material

Below is the link to the electronic supplementary material.

Characterization of CZS thin films produced by spin coating at 250 °C, specifics to chemical synthesis, experimental technique, and more analytical data (EDX, ICP-AES, p-XRD), band gap, lattice parameters, d-space, Raman, and resistivity) (DOCX 1553 kb)
