# Supplementary material for: The deposition of thin films of cadmium zinc sulfide Cd1−xZnxS at 250 °C from spin-coated xanthato complexes: a potential route to window layers for photovoltaic cells
Source: J Mater Sci. 2017 Dec 7;53(6):4360–70. doi: 10.1007/s10853-017-1872-1 (PMC6956951; doi:10.1007/s10853-017-1872-1)
Supplement: Supplementary file 1 — Characterization of CZS thin films produced by spin coating at 250 °C, specifics to chemical synthesis, experimental technique, and more analytical data (EDX, ICP-AES, p-XRD), band gap, lattice parameters, d-space, Raman, and resistivity) (DOCX 1553 kb) [file 10853_2017_1872_MOESM1_ESM.docx]

Supporting information

The deposition of thin films of cadmium zinc sulfide Cd_1-x_Zn_x_S at 250 °C from spin-coated xanthato complexes: a potential route to window layers for photovoltaic cells

Ali AK. Bakly^1[0000-0003-4163-2322]^, Ben F. Spencer^1[0000-0002-1453-5327]^ and Paul O’Brien^1,2,*[ 0000-0001-8744-9174]^

1 School of Materials, The University of Manchester, Oxford Road, M13 9PL

2 School of Chemistry, The University of Manchester, Oxford Road M13 9PL

**Table S1**. Mole% zinc in precursor mixture against alloy composition by EDX or ICP-AES (Cd1-xZnxS, 0 ≤ x ≤ 0.15) of thin films produced by spin coating precursors followed by decomposition at 250 °C.

| Mole% zinc in precursor | Cd | Zn | S | EDX | Cd | Zn | S | ICP |
| --- | --- | --- | --- | --- | --- | --- | --- | --- |
| 0 | 60.30 | 0 | 39.70 | 0 | 0.00659 | 0 | 0.01004 | 0 |
| 2.5 | 68.03 | 1.88 | 30.09 | 1.88 | 0.01301 | 0.00049 | 0.01523 | 1.70 |
| 5 | 70.18 | 2.26 | 27.57 | 2.26 | 0.00837 | 0.00047 | 0.01082 | 2.41 |
| 7.5 | 51.92 | 3.90 | 44.18 | 3.90 | 0.00772 | 0.00070 | 0.00970 | 3.88 |
| 10 | 69.77 | 4.36 | 25.87 | 4.36 | 0.00252 | 0.00038 | 0.00488 | 4.91 |
| 12.5 | 71.50 | 8.00 | 20.50 | 8.00 | 0.00814 | 0.00162 | 0.01070 | 7.92 |
| 15 | 51.29 | 7.29 | 41.41 | 7.29 | 0.00911 | 0.00168 | 0.01204 | 7.36 |

EDX and ICP analysis show that the films of alloy compositions for 0 ≤ x ≤ 0.15 are composed of cadmium: zinc: sulfur of the ratios placed in table S1.


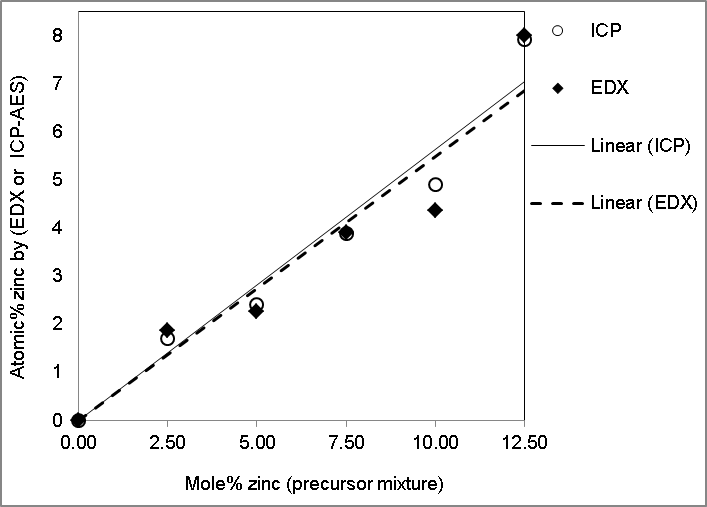


|  | Slope (b) | Intercept | R^2^ |  |  | Slope (b) | Intercept | R^2^ |
| --- | --- | --- | --- | --- | --- | --- | --- | --- |
| Value | 0.5609 | -0.1057 | 0.917 |  | Value | 0.5795 | -0.1509 | 0.957 |
| Std. error | 0.0840 | 0.6361 |  |  | Std. error | 0.0608 | 0.4606 |  |
| y= b*x + a | | EDX | |  | y= b*x + a | | ICP | |

**Fig S1.** Plot of mole% zinc in precursor mixture against alloy composition as determined by EDX or ICP-AES (Cd_1-x_Zn_x_S, 0 ≤ x ≤ 0.125) of thin films produced by spin coating precursors followed by decomposition at 250 °C.


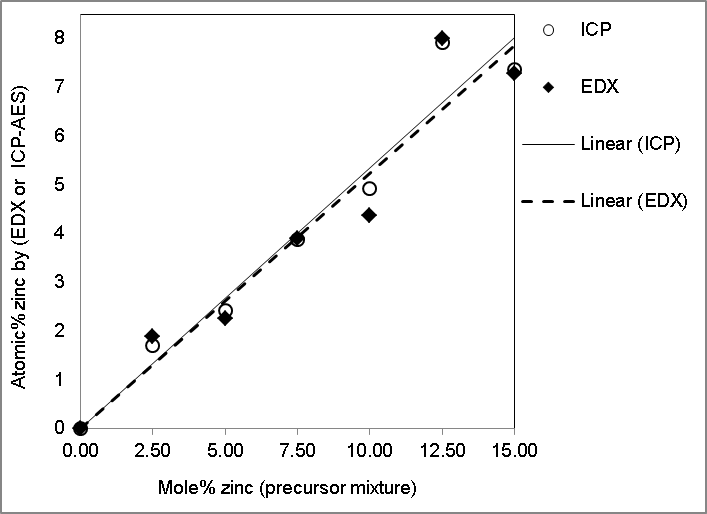


|  | Slope (b) | Intercept | R^2^ |  |  | Slope (b) | Intercept | R^2^ |
| --- | --- | --- | --- | --- | --- | --- | --- | --- |
| Value | 0.5173 | 0.0761 | 0.928 |  | Value | 0.5291 | 0.0593 | 0.954 |
| Std. error | 0.0645 | 0.5818 |  |  | Std. error | 0.0520 | 0.4683 |  |
| y= b*x + a | | EDX | |  | y= b*x + a | | ICP | |

**Fig S1.** Plot of mole% zinc in precursor mixture against alloy composition as determined by EDX or ICP-AES (Cd_1-x_Zn_x_S, 0 ≤ x ≤ 0.15) of thin films produced by spin coating precursors followed by decomposition at 250 °C.

The lattice parameters for the hexagonal structure were calculated from p-XRD Equation (a):

1/d^2^ = (4/3) [(h^2^ + hk + k^2^) / a^2^] + (l^2^/c^2^) (a)

**Table S2.** Summary table of mole% zinc in precursor mixture against the lattice constants (a and c) (Å) and unit cell volume (Å^3^) (Cd_1-x_Zn_x_S, 0 ≤ x ≤ 0.15) of thin films produced by spin coating precursors followed by decomposition at 250 °C.

| Phase | Mole% zinc in precursor | a / (Å) | c / (Å) | Unit cell vol. / (Å^3^) |
| --- | --- | --- | --- | --- |
| Hexagonal-CdS | 0% lit. [[1]] | 4.137 | 6.7144 | 99.5197 |
|  | 0 | 4.135 | 6.733 | 99.696 |
|  | 2.5 | 4.131 | 6.710 | 99.163 |
|  | 5 | 4.130 | 6.694 | 98.879 |
|  | 7.5 | 4.126 | 6.672 | 98.363 |
|  | 10 | 4.123 | 6.660 | 98.043 |
|  | 12.5 | 4.118 | 6.635 | 97.439 |
|  | 15 | 4.121 | 6.611 | 97.228 |
| Hexagonal-ZnS | 100% lit. [[2]] | 3.811 | 6.234 | 78.4107 |

Literature values at (x = 0 and x = 1).[1, 2]

**
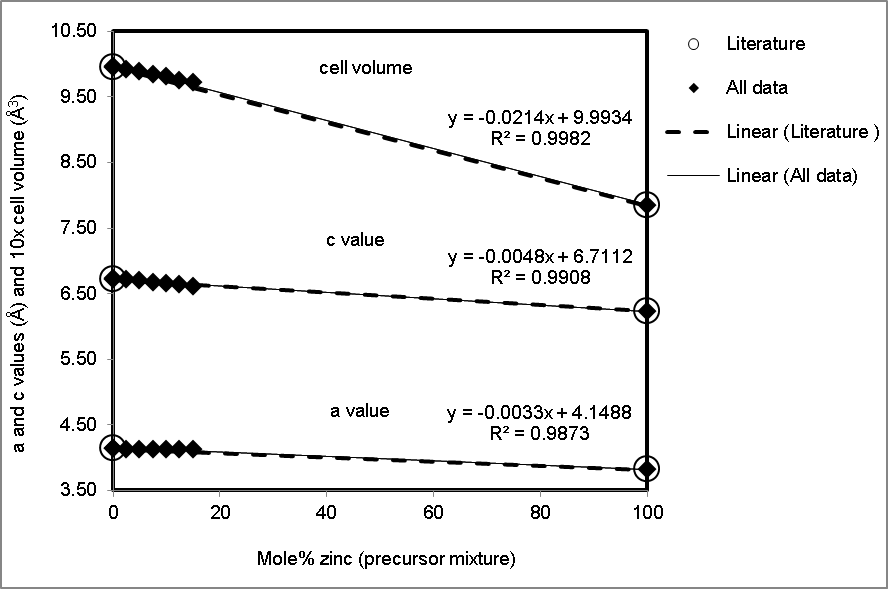
**

**Fig S3.** Mole% zinc in precursor mixture against the lattice constants (a and c) and unit cell volume (Cd_1-x_Zn_x_S, 0 ≤ x ≤ 0.15) of thin films produced by spin coating precursors followed by decomposition at 250 °C.

Literature values of the lattice constants (a and c) and unit cell volume at (x = 0 and x = 1) were indicated.[1, 2]


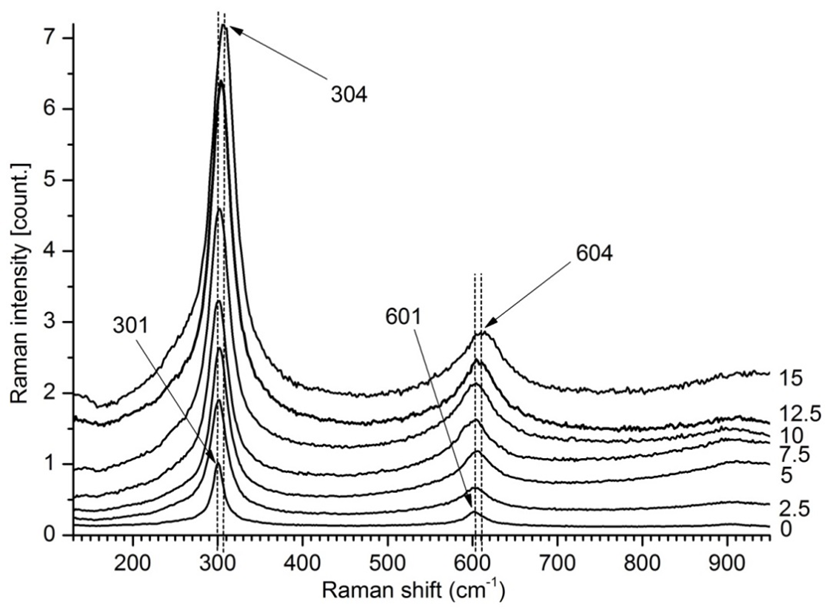


**Fig S4.** Raman spectrum of Cd_1-x_Zn_x_S thin films deposited in the composite range of (0 ≤ x ≤ 0.15) from spin coating (1) and (2) followed by decomposition at 250 °C.

**Table S3.** Mole% zinc in precursor mixture against Raman shift (Cd_1-x_Zn_x_S, 0 ≤ x ≤ 0.15) of thin films produced by spin coating precursors followed by decomposition at 250 °C.

| Mole% zinc in precursor | Raman shift |
| --- | --- |
| 0 | 301.12 |
| 2.5 | 301.49 |
| 5 | 302.24 |
| 7.5 | 302.53 |
| 10 | 303.09 |
| 12.5 | 303.57 |
| 15 | 304.51 |


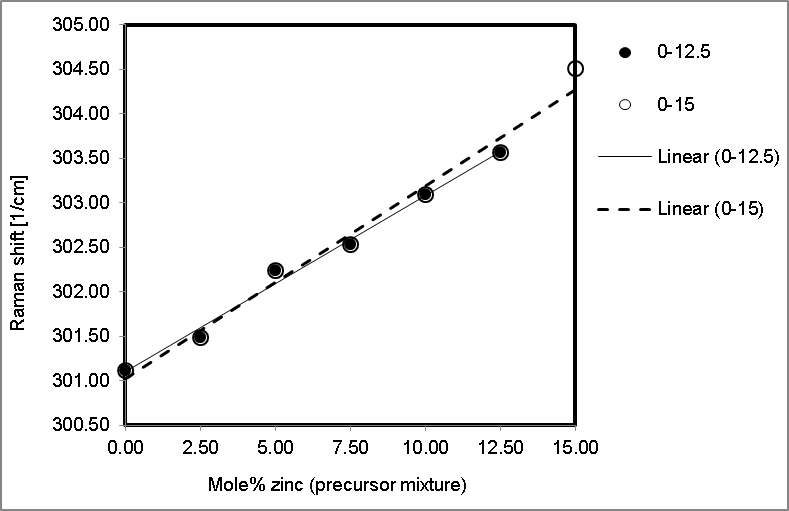


|  | Slope (b) | Intercept | R^2^ |  |  | Slope (b) | Intercept | R^2^ |
| --- | --- | --- | --- | --- | --- | --- | --- | --- |
| Value | 0.1982 | 301.101 | 0.9915 |  | Value | 0.2169 | 301.024 | 0.9834 |
| Std. error | 0 | 0 |  |  | Std. error | 0 | 0 |  |
| y= b*x + a | | (0-12.5) | |  | y= b*x + a | | (0-15) | |

**Fig S5.** Plot of Mole% zinc in precursor mixture against Raman shift (Cd_1-x_Zn_x_S, 0 ≤ x ≤ 0.125 and 0 ≤ x ≤ 0.15) of thin films produced by spin coating precursors followed by decomposition at 250.

**Table S4**. Mole% zinc in precursor mixture against optical band gap and d-exp (002) (Cd_1-x_Zn_x_S, 0 ≤ x ≤ 0.15) of thin films produced by spin coating precursors followed by decomposition at 250 °C for 1 h.

| Mole% zinc in precursor | Band gap (eV) | d-exp._002_ (Å) |
| --- | --- | --- |
| 0 | 2.34 | 3.3610 |
| 2.5 | 2.38 | 3.3465 |
| 5 | 2.41 | 3.3457 |
| 7.5 | 2.43 | 3.3378 |
| 10 | 2.455 | 3.3292 |
| 12.5 | 2.5 | 3.3168 |
| 15 | 2.51 | 3.3048 |


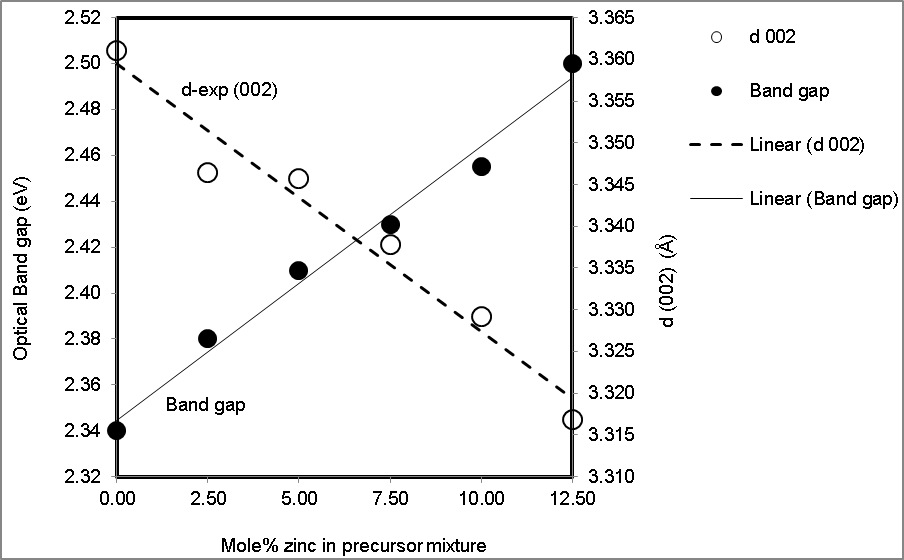


|  | Slope (b) | Intercept | R^2^ |  |  | Slope (b) | Intercept | R^2^ |
| --- | --- | --- | --- | --- | --- | --- | --- | --- |
| Value | 0.0119 | 2.3445 | 0.986 |  | Value | -0.0032 | 3.3595 | 0.967 |
| Std. error | 0.0007 | 0.0054 |  |  | Std. error | 0.0003 | 0.0025 |  |
| y= b*x + a | | Eg | |  | y= b*x + a | | d002 | |

**Fig S6**. Mole% zinc in precursor mixture against optical band gap and d-exp. (002) (Cd_1-x_Zn_x_S, 0 ≤ x ≤ 0.125) of thin films produced by spin coating precursors (1) and (2) followed by decomposition at 250 °C for 1 h.


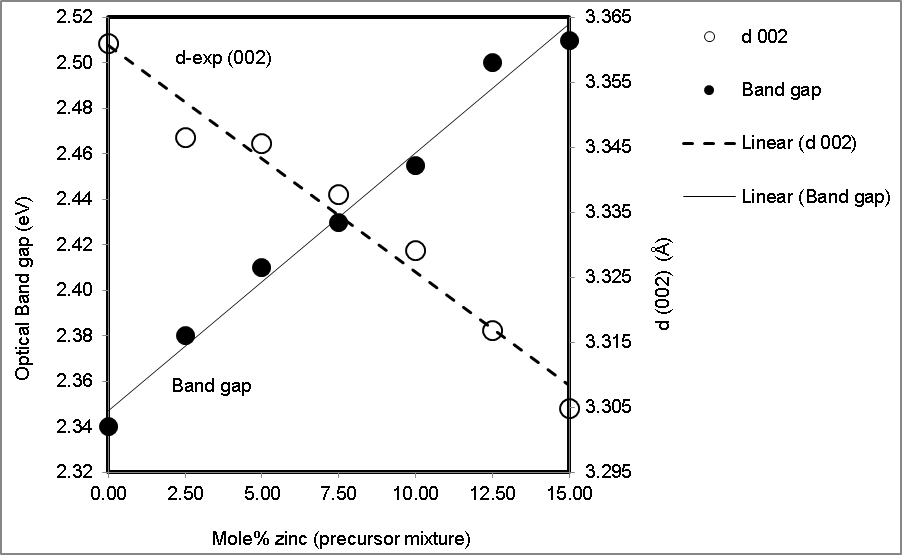


|  | Slope (b) | Intercept | R^2^ |  |  | Slope (b) | Intercept | R^2^ |
| --- | --- | --- | --- | --- | --- | --- | --- | --- |
| Value | 0.0114 | 2.3470 | 0.986 |  | Value | -0.0035 | 3.3607 | 0.968 |
| Std. error | 0.0006 | 0.0055 |  |  | Std. error | 0.0003 | 0.0026 |  |
| y= b*x + a | | Eg | |  | y= b*x + a | | d002 | |

**Fig S7**. Mole% zinc in precursor mixture against optical band gap and d-exp. (002) (Cd_1-x_Zn_x_S, 0 ≤ x ≤ 0.15) of thin films produced by spin coating precursors (1) and (2) followed by decomposition at 250 °C for 1 h.

The Stern relation (Equation b) estimates the optical band gap for near edge absorption,[3] when [v is the frequency, h is the Planck’s constant; k equals a constant, α, is the absorbance while n carries the value of either (1 or 4) for the direct and indirect transition, respectively]:

$\alpha=\frac{[k\left( hv-Eg \right)]^{n/2}}{\mathrm{hv}}$ (b)


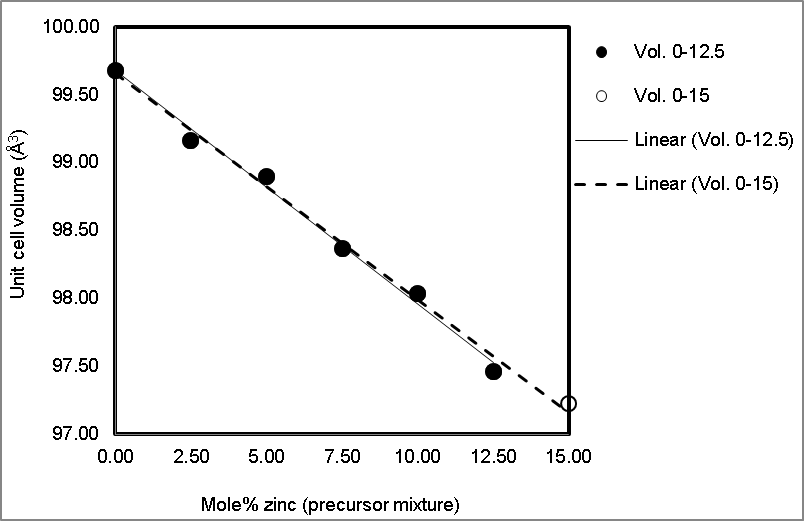


|  | Slope (b) | Intercept | R^2^ |  |  | Slope (b) | Intercept | R^2^ |
| --- | --- | --- | --- | --- | --- | --- | --- | --- |
| Value | -0.1714 | 99.6736 | 0.993 |  | Value | -0.1661 | 99.6512 | 0.993 |
| Std. error | 0.0075 | 0.0565 |  |  | Std. error | 0.0061 | 0.0552 |  |
| y= b*x + a | | (0-12.5) | |  | y= b*x + a | | (0-15) | |

**Fig S8**. Mole% zinc in precursor mixture against unit cell volume (Cd_1-x_Zn_x_S, 0 ≤ x ≤ 0.125 and 0 ≤ x ≤ 0.15) of thin films produced by spin coating precursors (1) and (2) followed by decomposition at 250 °C for 1 h.

**Table S5**. Mole% zinc in precursor mixture against the measured resistivity (Cd_1-x_Zn_x_S, 0 ≤ x ≤ 0.15) of thin films produced by spin coating precursors (1) and (2) followed by decomposition at 250 °C for 1 h.

| Mole% zinc in precursor | Resistivity*10^^5^ (Ω.cm) |
| --- | --- |
| 0 | 0.5801 |
| 2.5 | 0.7251 |
| 5 | 0.7553 |
| 7.5 | 0.7795 |
| 10 | 0.9245 |
| 12.5 | 1.1179 |
| 15 | 1.2085 |


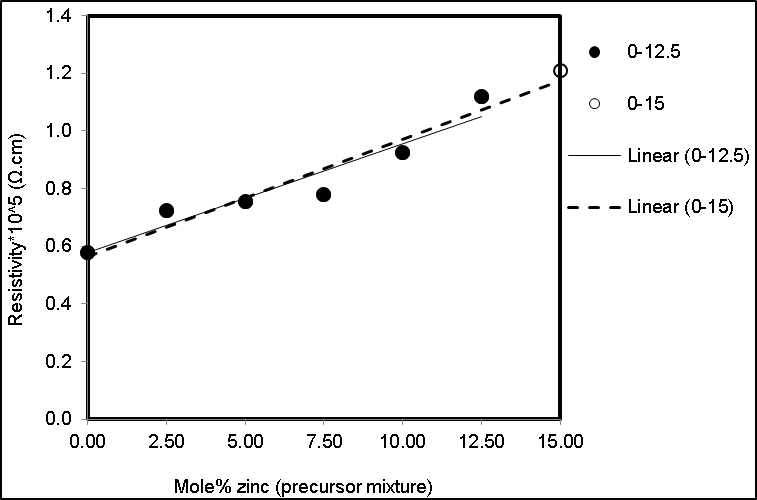


|  | Slope (b) | Intercept | R^2^ |  |  | Slope (b) | Intercept | R^2^ |
| --- | --- | --- | --- | --- | --- | --- | --- | --- |
| Value | 0.0378 | 0.5772 | 0.912 |  | Value | 0.0406 | 0.5659 | 0.943 |
| Std. error | 0.0059 | 0.0446 |  |  | Std. error | 0.0045 | 0.0401 |  |
| y= b*x + a | | (0-12.5) | |  | y= b*x + a | | (0-15) | |

**Fig S9**. Mole% zinc in precursor mixture against the measured resistivity (Cd_1-x_Zn_x_S, 0 ≤ x ≤ 0.125 and 0 ≤ x ≤ 0.15) of thin films produced by spin coating precursors (1) and (2) followed by decomposition at 250 °C for 1 h.


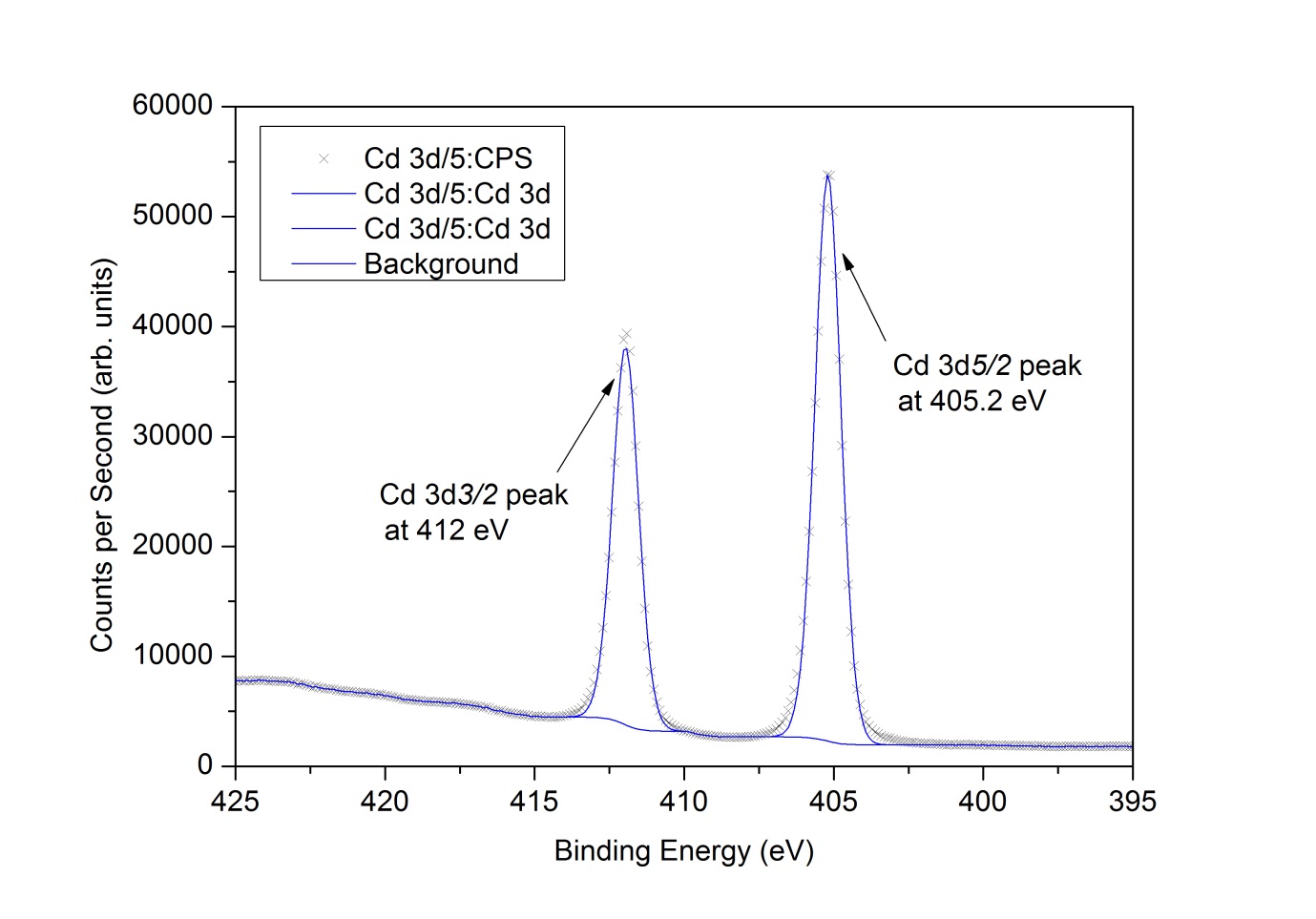


**Fig S10**. XP narrow scan spectrum shows a single chemical species for Cd 3d doublet photoelectron peak for (CdS) thin film.


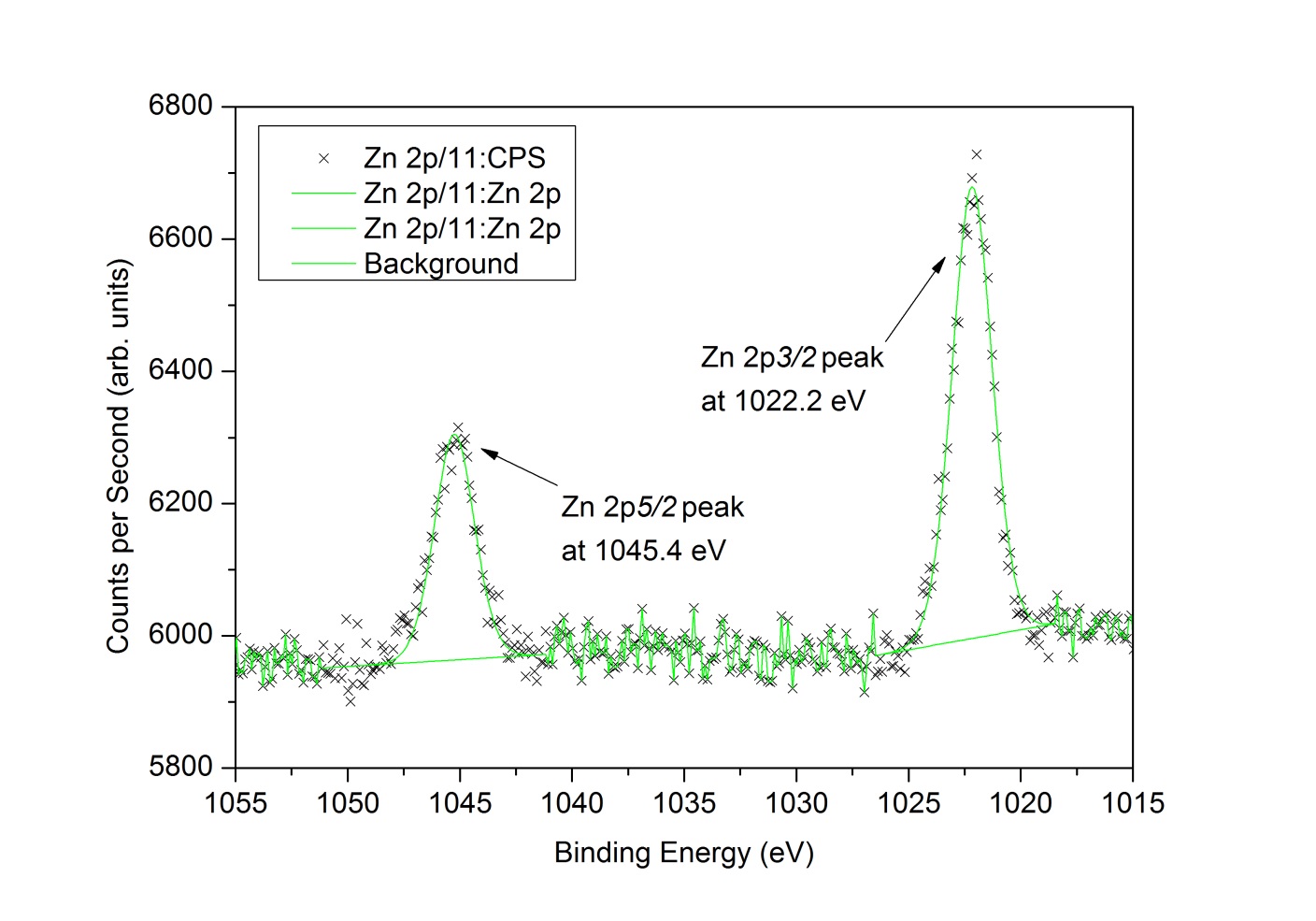


**Fig S11**. XP narrow scan spectrum shows a single chemical species for Zn 2p peak for (ZnS) thin film.


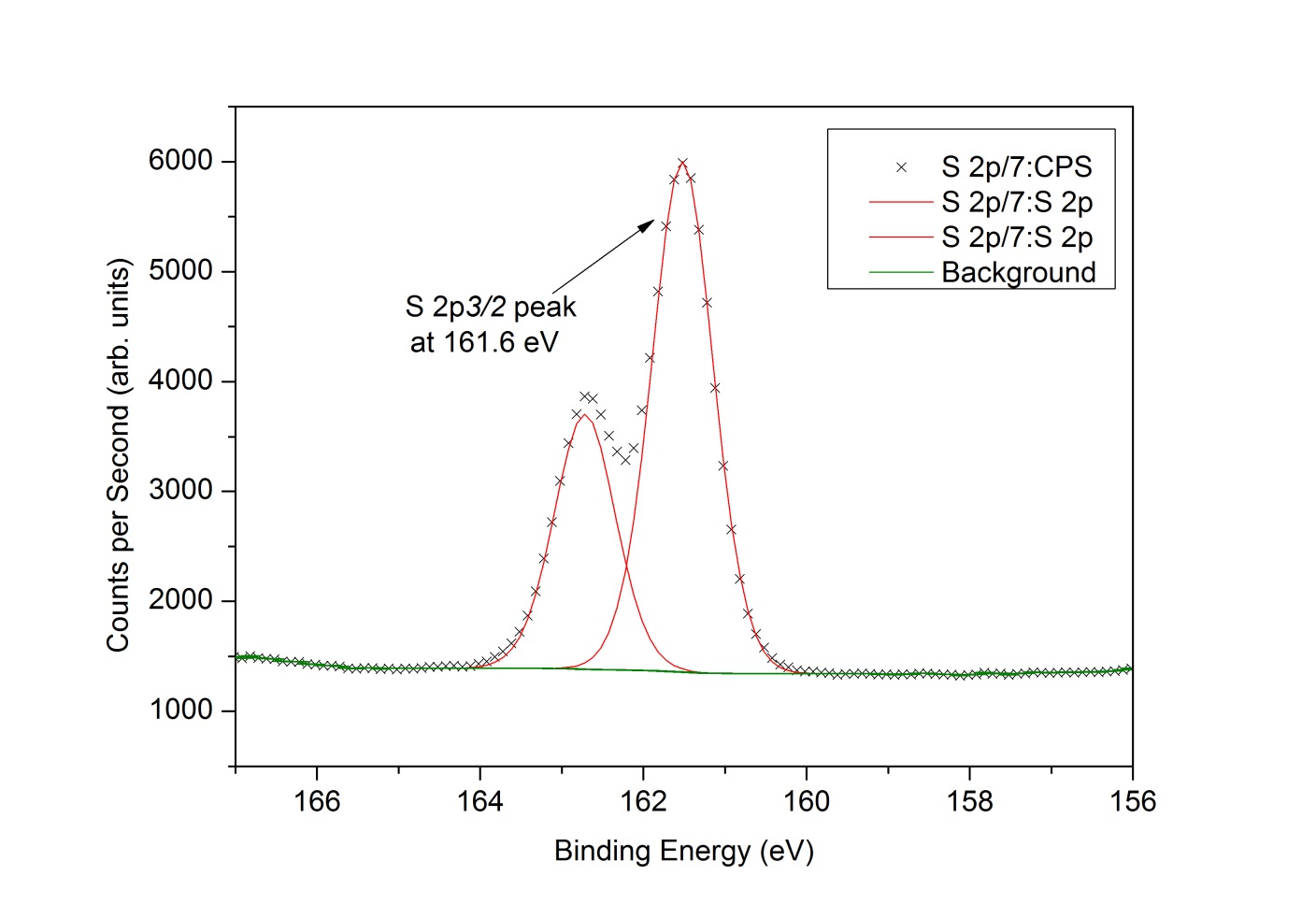


**Fig S12**. XP narrow scan spectrum for S 2p photoelectron peak at a binding energy position of 161.6 eV associated with CdS and ZnS (same electronegativity).

Both of the binding energy (BE) values and the distance between the S2p and Zn 2p_3/2_, lines vary from work to work. The main reasons are probably differences in conductivity of the samples due to impurities and partial compensation of charging due to differences in experimental conditions. XPS literature values were indicated; Cd 3d peak for (CdS) thin film,[4] Zn 2p peak for (ZnS) thin film,[5] and S 2p photoelectron peak associated with CdS,[4] and ZnS.[5]

**Table S6**. Lattice mismatch calculation for the buffer materials and CdZnS.

The literature values of lattice constants for buffer materials that placed in Table S8 have been indicated under each category.[6–8] † (á) is the average separation of atoms in the “close packed” 111 plane. ‡ Lattice constants range for Cd_1-x_Zn_x_S is 4.135-4.121 Å.


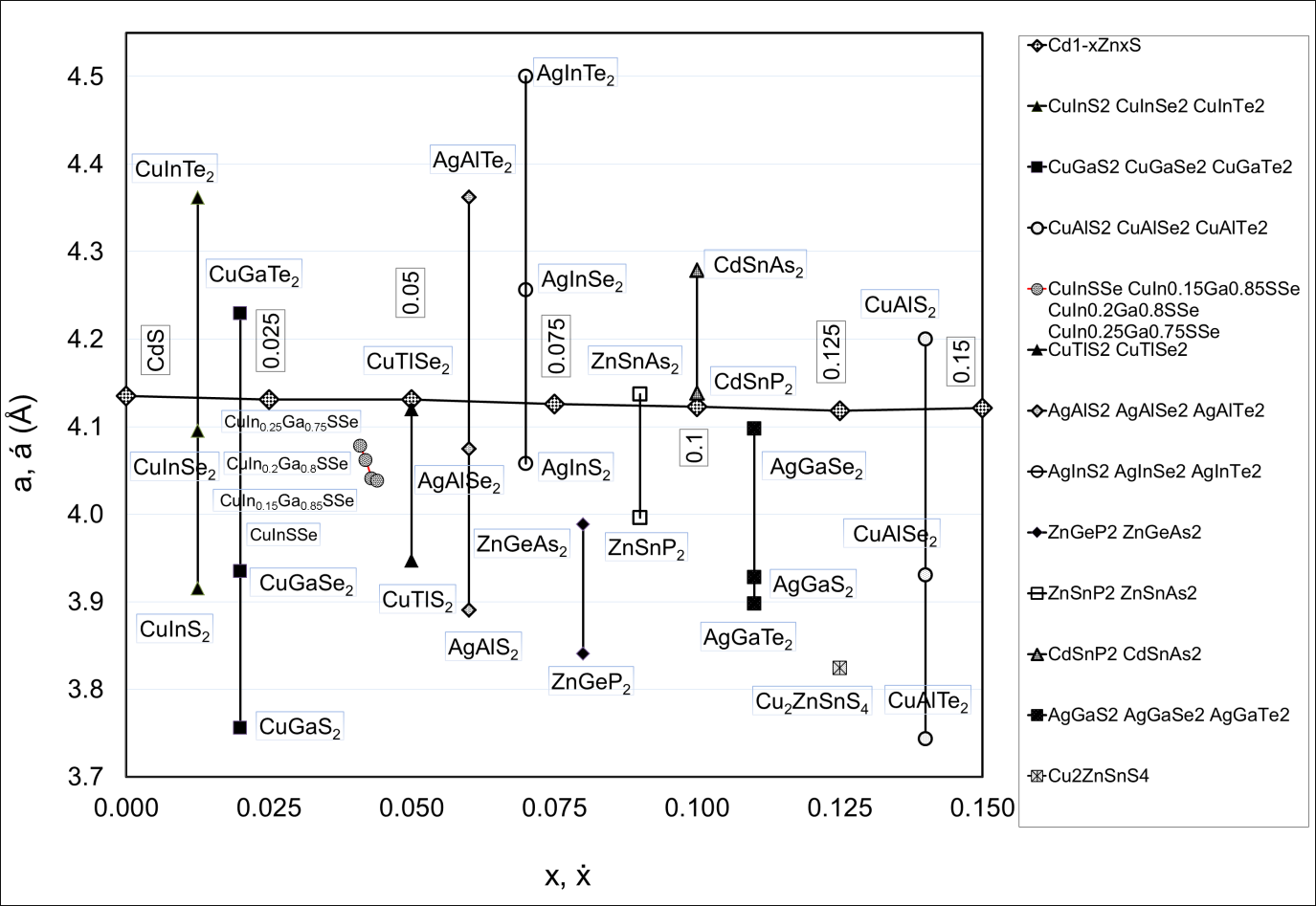


**Fig S13**. Buffer materials and Cd_1-x_Zn_x_S lattice mismatch.

The literature values for the lattice parameters of the buffer materials were provided.[6–8]


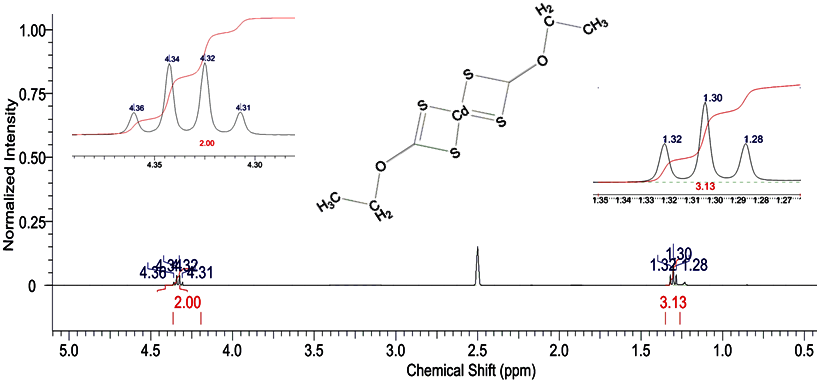


**Fig S14**. Proton NMR spectra for cadmium ethyl xanthato precursor (400 MHz, DMSO-d6).


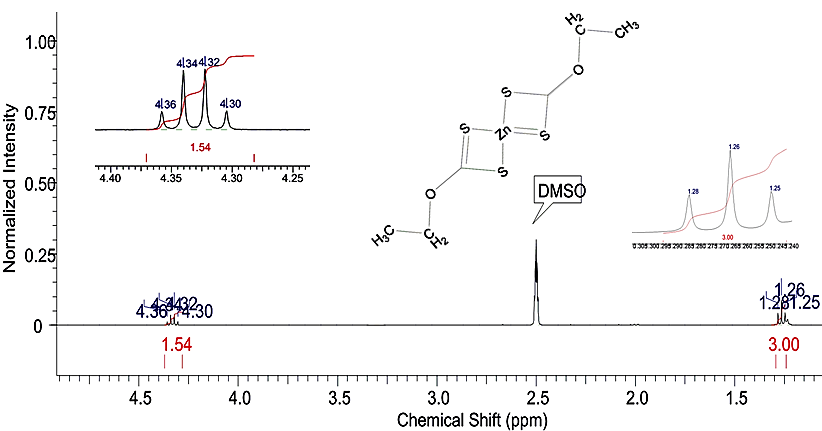


**Fig S15**. Proton NMR spectra for zinc ethyl xanthato precursor (400 MHz, DMSO-d6).

The H_2_O peak in proton NMR spectra has been smoothed.


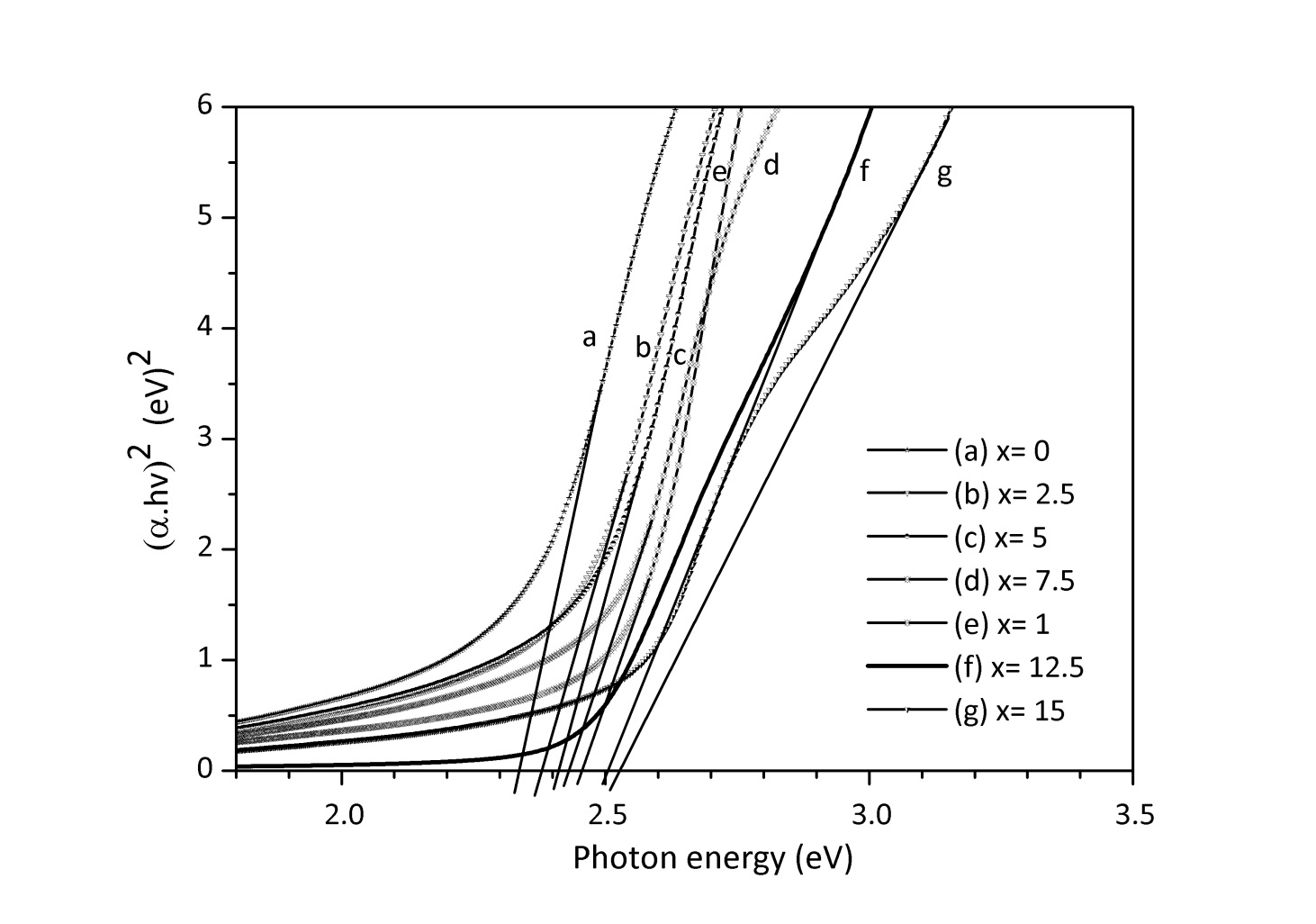


**Fig. S16** The typical Tauc plots showing extrapolation of the absorption spectrum to obtain the optical band gap for (0-15) mole% zinc films deposited from (1) and (2) spin coated on a glass substrate, (250 °C for 1 h) under N^2^ gas stream.


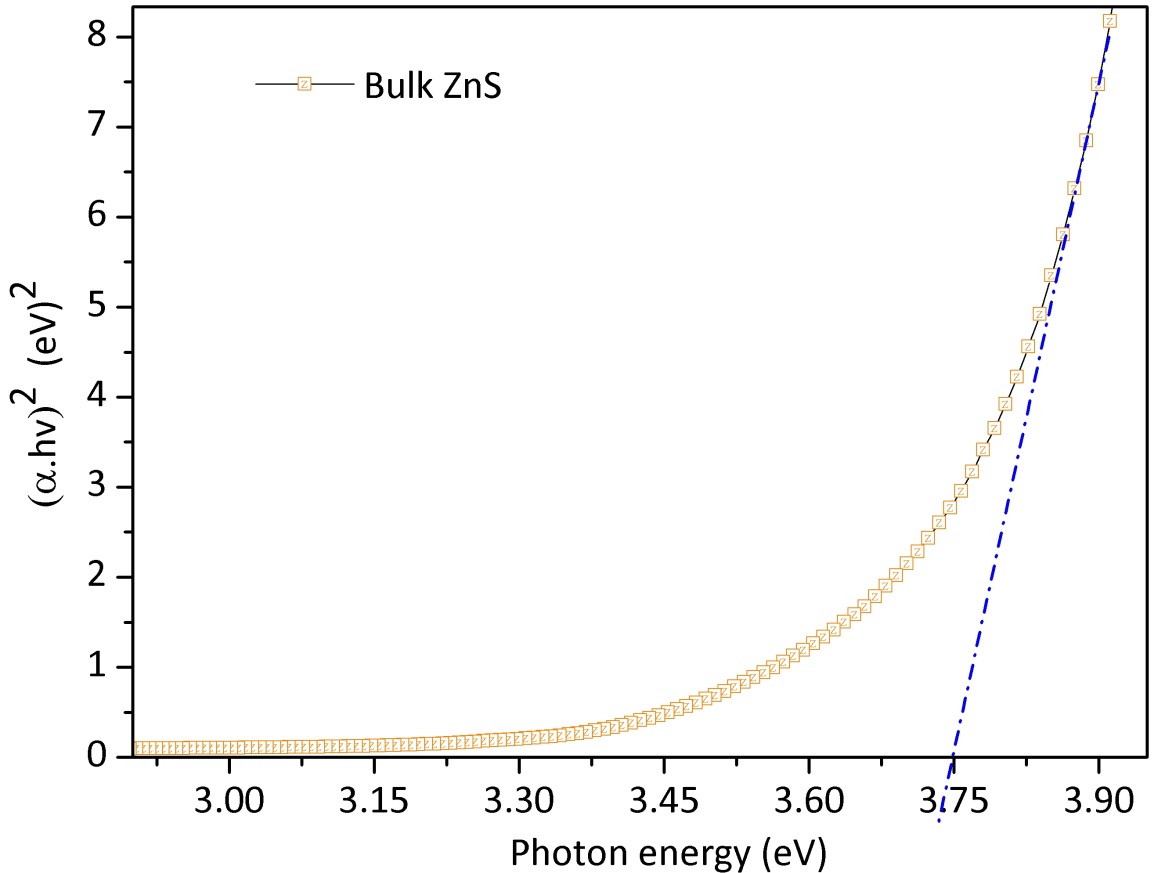


**Fig. S17** A typical Tauc plot showing extrapolation of the absorption spectrum to obtain the optical band gap for the bulk zinc film deposited from (2) spin coated on a glass substrate, (250 °C for 1 h) under N^2^ gas stream.

REFERENCES

1. Fe F, Bo M (2005) Physical Properties : Chemistry : Nat Hist 2:16383. doi: 10.3746/jfn.2005.10.2.187

2. Wyckoff R W G. (1963) Crystal Structures, second edition. Interscience Publishers, New York, New York.

3. Kassim A, WeeTee T (2010) Preparation and Characterization of Iron Sulphide Thin Films By Chemical Bath Deposition Method. Indo J Chem 10:8–11.

4. Marychurch M, Morris GC (1985) X-ray photoelectron spectra of crystal and thin film cadmium sulphide. Surf Sci 154:251–254. doi: 10.1016/0039-6028(85)90035-4

5. Laajalehto K, Kartio I, Nowak P (1994) XPS study of clean metal sulfide surfaces. Appl Surf Sci 81:11–15. doi: 10.1016/0169-4332(94)90080-9

6. Jaffe JE, Zunger A (1984) Theory of the band-gap anomaly in AB C 2 chalcopyrite semiconductors. Phys Rev B 29:1882.

7. Hossain MA, Tianliang Z, Keat LK, et al (2015) Synthesis of Cu (In, Ga)(S, Se) 2 thin films using an aqueous spray-pyrolysis approach, and their solar cell efficiency of 10.5%. J Mater Chem A 3:4147–4154.

8. Seboui Z, Cuminal Y, Kamoun-Turki N (2013) Physical properties of Cu2ZnSnS4 thin films deposited by spray pyrolysis technique. J Renew Sustain Energy 5:23113.
